# Antimicrobial Resistance in Oral Healthcare: A Growing Concern in Dentistry

**DOI:** 10.3390/dj13090391

**Published:** 2025-08-28

**Authors:** Golnoush Farzinnia, Yalda Anvari, Michelle F. Siqueira

**Affiliations:** College of Dentistry, University of Saskatchewan, Saskatoon, SK S7N 5A2, Canada; ogk610@mail.usask.ca (G.F.); drc263@mail.usask.ca (Y.A.)

**Keywords:** biofilms, dental unit waterlines, dentistry, dentures, drug resistance, microbial, orthodontic appliances

## Abstract

One of the most significant public health issues of the twenty-first century is antimicrobial resistance (AMR), which is responsible for thousands of deaths each year and undermines the efficacy of commonly used antibiotics. In dentistry, the frequent and sometimes inappropriate use of antibiotics, combined with the complexity of the oral microbiome, creates a high-risk environment for the development and spread of antimicrobial-resistant microorganisms. Although clinical infections have received most of the attention, dental unit waterlines, dentures, and orthodontic appliances are three reservoirs that play an important but underestimated role in this global crisis. These environments provide perfect conditions for biofilm formation and, further, the survival and growth of resistant microorganisms. This review aims to discuss the origins and mechanisms of AMR, the unrecognized role of these three reservoirs in dental settings, and their contribution to the AMR issue. It also highlights the necessity of strict infection control procedures and targeted antimicrobial stewardship strategies to overcome this growing threat.

## 1. Introduction

Antimicrobial resistance (AMR) has arisen as one of the most concerning public health concerns of the 21st century. AMR poses a threat to the effective treatment and prevention of a growing number of infections caused by bacteria, parasites, viruses, and fungi that are no longer susceptible to the antibiotics [1]. AMR was directly responsible for 1.27 million deaths in 2019 and was linked to approximately 5 million deaths globally [2]. According to one systematic analysis in 2022, the highest mortality rates are in western sub-Saharan Africa and South Asia, and the lowest in Australasia [3]. *Escherichia coli*, *Staphylococcus aureus*, *Klebsiella pneumoniae*, *Streptococcus pneumoniae*, *Acinetobacter baumannii*, and *Pseudomonas aeruginosa* caused nearly three-quarters of attributable deaths, mainly from lower respiratory, bloodstream, and intra-abdominal infections [3]. Resistance to β-lactams and fluoroquinolones accounted for over 70% of the burden, with the greatest impact in low-resource settings [3]. The UK government-commissioned review concluded that AMR may kill 10 million people annually by 2050 [4,5]. According to WHO and academic literature, AMR is a serious issue that must be addressed with a coordinated and worldwide plan [1,6,7,8].

In dental practice, antibiotics are typically prescribed to treat both non-odontogenic and odontogenic infections, as well as prophylactically in high-risk patients, such as those with systemic conditions such as endocarditis or congenital heart disease, to prevent focal infections. Antibiotics are also prescribed to prevent localized, systemic, and surgical site infections in patients undergoing oral surgery or dental procedures [9]. Dentistry, whether for therapeutic or prophylactic uses, accounts for almost 10% of global antibiotic prescriptions. However, inappropriate antibiotic prescribing could lead to misuse or overuse of antibiotics in dental practice [9,10]. Additionally, patient non-adherence to prescribed antimicrobial treatments further exacerbates the misuse and overuse of antibiotics in dentistry [11]. Consequently, AMR in dentistry remains a significant public health issue [12].

Several domains within dentistry contribute to the challenge of AMR. Previous systematic reviews have covered AMR in antibiotic prophylaxis in dental implants and tooth extractions, odontogenic infections, and periodontal diseases. A systematic review and meta-analysis of antibiotic prophylaxis in dental implants and extractions highlighted the risk of accelerating AMR through unnecessary prescriptions [13]. Other reviews have focused on odontogenic infections and reported resistance to antibiotics such as penicillin, clindamycin, and amoxicillin among *Staphylococcus*, *Streptococcus*, *Klebsiella*, *and Enterococcus* [14,15]. Another systematic review of AMR in periodontal diseases indicated that periodontal pathogens could develop resistance to popular antibiotics such as metronidazole, clindamycin, and amoxicillin [16].

**Figure 1:**
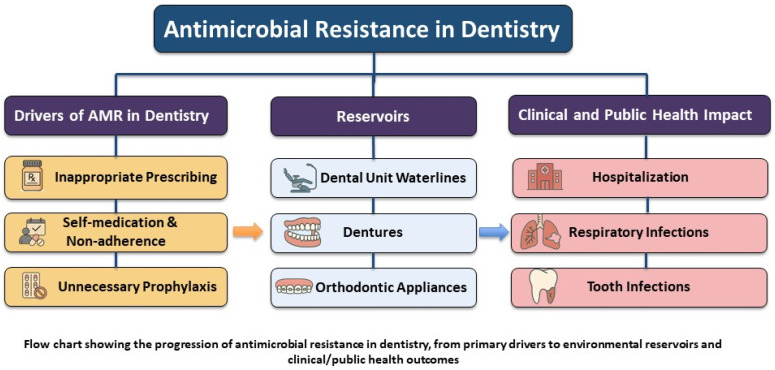
Overall scope of AMR in dentistry.

There are three overlooked but significant reservoirs of antibiotic-resistant bacteria in dental settings: dental unit waterlines (DUWLs), dentures, and orthodontic appliances (Figure 1) [17,18,19]. Persistent biofilms are maintained in these environments, offering a safe niche for resistant microorganisms to survive, evolve, and spread [17,18,19]. Such environments call for targeted infection control measures and antimicrobial stewardship (AMS) strategies to mitigate the risk of resistance transmission in dental settings. AMS in dentistry is in its early stages, with no systematic, coordinated, or consensus-driven approach [20]. Moreover, there is a gap in research, particularly in the context of non-infectious reservoirs such as DUWLs, dentures, and orthodontic appliances, which are often underestimated in current AMS strategies. Regarding the growing threat of AMR, further investigation is needed to determine how well current infection control and AMS strategies address the potential role of these reservoirs in the development and spread of resistant microorganisms.

Thus, this narrative review aims to highlight the role of DUWLs, dentures, and orthodontic appliances as overlooked reservoirs of antibiotic-resistant microorganisms and to determine the need for targeted infection control measures and AMS strategies to address these critical but under-researched areas in dental settings. Relevant articles were selected based on their relevance and contribution to the topic. As this is a narrative review, no formal risk of bias or quality assessment was conducted.

## 2. Origins of Antimicrobial Resistance

**Figure 2:**
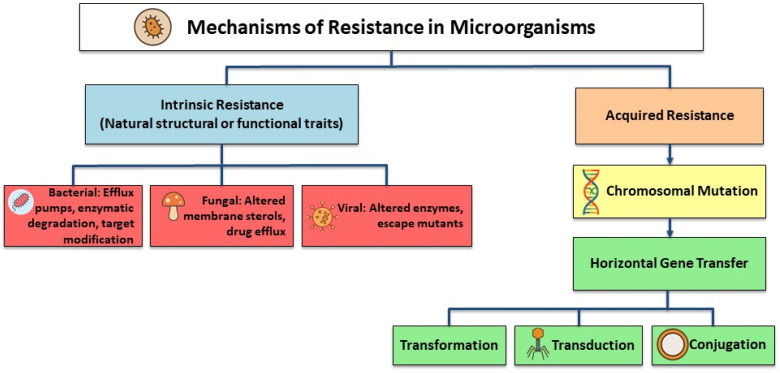
Primary AMR mechanisms.

There are two types of AMR: intrinsic (natural) resistance and acquired resistance. Intrinsic resistance is a common trait shared by all strains of a particular species of microorganism, arises independently of previous antibiotic exposure, and is unrelated to horizontal gene transfer (Figure 2) [21,22]. The main bacterial mechanisms that contribute to intrinsic resistance are the outer membrane’s decreased permeability, particularly due to the presence of lipopolysaccharide (LPS) in Gram-negative bacteria, and the natural activity of efflux pumps, which actively expel antimicrobial agents from the cell [21].

On the other hand, acquired resistance does not affect the entire species of bacteria; it only affects specific strains. There are two mechanisms for acquired resistance. The first is chromosomal mutation, where changes to the microorganism’s DNA occur spontaneously, whether the antibiotics were used or not [23]. For instance, chromosomal mutations in bacteria can alter the penicillin-binding proteins (PBPs), which are the site of action for penicillin antibiotics, giving the bacteria resistance to the antibiotic [24].

The second mechanism of acquired resistance is horizontal gene transfer, which allows resistant genes to spread between different bacterial species and strains of the same species [25]. Horizontal gene transfer is one of the most concerning mode of resistance [23], with three mechanisms: transformation, transduction, and conjugation [24]. Transformation is when bacteria with antibiotic resistance genes (ARGs) release pieces of their DNA into the environment, where they can be absorbed by nearby bacteria. Transduction follows similar steps, except that a phage particle containing the DNA segment with the resistance genes delivers the genes to the recipient bacteria. Conjugation is the direct transfer of a plasmid from a donor bacterium to a recipient through physical contact [26].

Since chromosomal mutations are limited by the bacterial life cycle, horizontal gene transfer allows bacteria to develop antibiotic resistance more rapidly. However, horizontal gene transfer enables resistance genes to proliferate among bacteria regardless of their growth rate. Conjugation is considered the most common route of horizontal gene transfer, allowing genetic material to be transferred directly between bacterial cells. Both in human populations and in the environment, this mechanism plays a major role in the spread of genes that confer resistance to antibiotics and the development of multi-drug-resistant bacteria [23].

## 3. Mechanisms of Resistance

The four primary categories of AMR mechanisms are (1) active drug efflux, (2) inactivating a drug, (3) limiting drug uptake, and (4) modifying the drug target.

- **Active drug efflux**: Energy-dependent transport proteins, such as those from the ATP-binding cassette (ABC) family or major facilitator superfamily (MFS), pump antimicrobial agents out of the cell before they reach their target concentration [27]. In Gram-negative bacteria, efflux systems like AcrAB-TolC can expel multiple classes of antibiotics, leading to multi-drug resistance [28].
- **Drug inactivation**: Bacteria produce enzymes that chemically modify or destroy the antimicrobial agent. Classic examples include β-lactamases, which hydrolyse the β-lactam ring in penicillins and cephalosporins, and aminoglycoside-modifying enzymes that acetylate, phosphorylate, or adenylate the drug, making it ineffective [29].
- **Limiting drug uptake**: Alterations in outer membrane porins or cell wall structure reduce the permeability of the bacterial cell envelope, and as a result, it can prevent antibiotics from entering. This is common in Gram-negative bacteria, where porin loss or modification limits the uptake of β-lactams and fluoroquinolones [30].
- **Target modification**: Genetic mutations or enzymatic alterations change the binding site of the drug so that it no longer interacts effectively [31]. For instance, mutations in PBPs confer resistance to β-lactams, while alterations in DNA gyrase or topoisomerase II lead to fluoroquinolone resistance [32].

Due to the outer membrane and efflux capabilities in Gram-negative bacteria, these bacteria can use all four main mechanisms. On the other hand, Gram-positive bacteria have fewer types of efflux pumps and less commonly limit drug uptake [33].

Mechanisms of antifungal resistance are expelling antifungal agents via efflux pumps (ABC and MFS transporters), enzymatic drug inactivation, modifying drug targets such as ergosterol biosynthesis (*ERG11* mutations in azole resistance), and reduced drug uptake by altering membrane composition [34]. Viruses develop resistance to antiviral drugs and vaccines primarily through random mutations, which can change important viral enzymes, including proteases or polymerases. Furthermore, segmented viruses, such as influenza, can develop resistance through genetic reassortment, which involves exchanging gene segments with other viral strains [35]. Although the mechanism of resistance used by bacteria, fungi, and viruses differ due to structural and metabolic variations, they are all contributors to the problem of AMR.

## 4. Antimicrobial Resistance in Dental Settings

AMR is a growing concern within the dental field [36]. The oral cavity hosts a wide range of microorganisms, many of which exist in biofilms. Consequently, the oral cavity creates a perfect environment for the spread of antibiotic resistance through horizontal gene transfer [37].

The frequent use of prophylactic antibiotics in extraction and implant procedures is one of the root causes of AMR in dentistry. Antibiotics are prescribed for high-risk patients; however, they are frequently given to healthy people unnecessarily, which leads to oral bacterial resistance [13]. Due to selective pressure from misuse, common medicines such as amoxicillin are no longer as effective against *Streptococcus* spp. and anaerobic bacteria [38,39,40].

Beyond surgical prophylaxis, empirical antibiotic therapy without bacterial culture and susceptibility testing is commonly used to treat odontogenic infections, including periapical abscesses. Key pathogens such as *Prevotella* spp., *Fusobacterium* spp., and *Streptococcus* spp. have become more resistant, with some strains acquiring beta-lactamase-mediated resistance to amoxicillin and penicillin [40,41,42]. When once-effective antibiotics fail, dentists have to turn to second-line options, which can accelerate the resistance trends [43].

In dental plaques, a wide range of bacterial species can share and acquire resistance genes [44,45]. While antibiotics and scaling are used together to manage periodontal diseases, this approach could unintentionally promote the survival and proliferation of resistant strains such as *Aggregatibacter actinomycetemcomitans* and *Porphyromonas gingivalis* [46].

**Figure 3:**
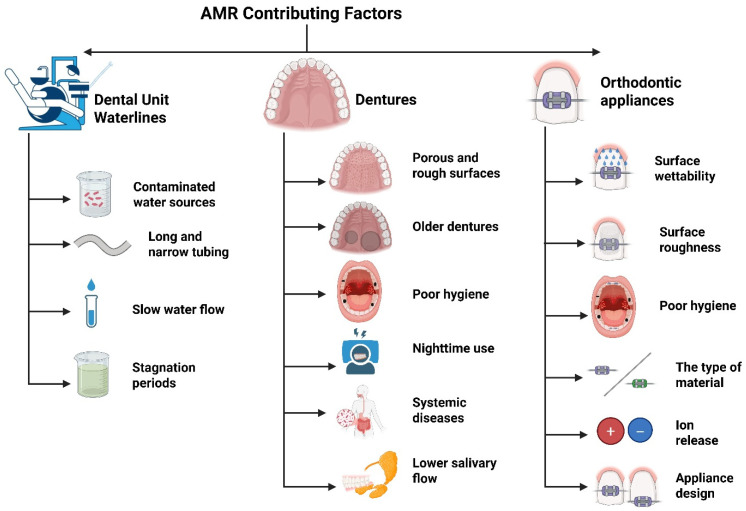
Major AMR contributors in DUWLs, dentures, and orthodontic appliances.

Although much of the current research on AMR in dentistry has focused on improper antibiotic use and clinical infections, other factors may also play a role. For instance, DUWLs, dentures, and orthodontic appliances create ideal conditions for biofilm formation and the spread of ARGs and contribute to the broader AMR problem. Since these areas are frequently exposed to oral microorganisms and may not be subjected to adequate, regular cleaning or maintenance protocols, resistant strains can survive and proliferate. Thus, it is crucial to investigate the role these environments play in the broader context of AMR and determine if targeted strategies are required to address them. Figure 3 summarizes the major factors contributing to AMR in these environments.

### 4.1. Antimicrobial Resistance in DUWLs

#### 4.1.1. Biofilm Formation in DUWLs and Its Role in Antimicrobial Resistance

Dental unit waterlines (DUWLs) are a complex system of tubes that supply water to rotating tools and ultrasonic scalers to cool the teeth and instruments during dental procedures and irrigate the operative site [47]. This system comprises valves, connectors, and approximately 6 m of plastic tubing usually made of polyvinyl or polyurethane, with internal diameters ranging from 0.5 to 2 mm [47]. Within this system, the maximum speed of water flow occurs at the center and gradually decreases toward the periphery and finally reaches zero at the tube walls [47]. Microorganisms contaminating DUWLs often originate from the municipal water supply [48]. Additionally, patient-derived sources, such as the retrograde flow of saliva into the waterlines, facilitate the entry of oral microbes into the system. This backflow often occurs at the end of the use of rotary dental instruments use, when the cessation or reduction of water flow creates a suction effect [49,50].

There are some contributing factors that facilitate biofilm formation inside DUWLs [51]. The tubes are long and narrow, and the slow water flow, typical of many dental procedures, creates a perfect environment for microbial buildup. Periods of stagnation, such as overnight, on weekends, or during holidays when dental clinics are closed, further promote biofilm formation [52,53]. These biofilms consist of microorganisms such as bacteria, fungi, protozoa, and viruses, all embedded in an extracellular matrix of polysaccharides and proteins [54].

Microorganisms within a biofilm are more difficult to remove than free-floating (planktonic) bacteria since they are more resistant to both antibiotics and the host’s immune system [55]. As a result, eliminating a mature biofilm often requires significantly higher doses of antimicrobial agents. Only a small number of cells in the biofilm are directly exposed to the antibiotic, since the surrounding exopolysaccharide matrix acts as a barrier, limiting the drug’s ability to penetrate the deeper layers [56]. The effective concentration of some antibiotics against biofilm-associated bacteria can be up to 100 to 1000 times higher [57]. Furthermore, the bacteria in biofilms are usually sessile, meaning they grow and divide very slowly due to their low metabolic activity [58]. Therefore, antibiotics that target actively dividing cells are often ineffective. Further, in biofilms, the close physical arrangement of bacterial cells may promote the horizontal transfer of genes, indicating that ARGs may be more easily shared in these bacterial communities [55,59,60].

#### 4.1.2. Common Microorganisms in DUWLs

Tap water contains low levels of opportunistic pathogens such as *S. aureus*, *P. aeruginosa*, *Legionella pneumophila*, *Mycobacterium tuberculosis*, and *nontuberculous mycobacteria* (NTM); biofilms inside DUWLs can provide a favorable environment for these microorganisms to multiply [61,62,63]. Water samples from DUWLs have been found to contain a variety of microorganisms, including *L. pneumophila*, *P. aeruginosa*, *Staphylococcus* spp., *Streptococcus* spp., *Enterobacter* spp., *Enterococcus* spp., viruses, and fungi [50,64,65].

The water from DUWLs also forms aerosols containing these microorganisms during procedures; these aerosols can be inhaled by dental staff and patients. During other procedures, water from DUWLs comes into direct contact with surgical sites in the oral cavity. Thus, the microbiological quality of water from DUWLs is critically important [61]. Contamination of DUWLs poses a potential health risk, particularly for vulnerable groups such as the elderly, children, immunocompromised individuals, pregnant women, smokers, and individuals with chronic diseases. Additionally, dental personnel are frequently exposed to aerosols and water from DUWLs during clinical procedures, placing them at occupational risk of infection, even if they are otherwise healthy [61,66]. One of the most significant cases is the 1995 death of a dentist from pneumonia caused by *Legionella dumoffii*, which was isolated from a dental unit in California [67]. Furthermore, some studies have revealed that dental professionals have significantly higher antibodies against *L. pneumophila* than a control group [68,69]. There have also been suspected fatal cases of legionellosis to have originated in dental settings [70,71].

While some of these microorganisms show low pathogenicity, others could be opportunistic and are among the main contributors to hospital-acquired infections. The ‘ESKAPE’ pathogens (*Enterococcus faecium*, *S. aureus*, *K. pneumoniae*, *A. baumannii*, *P. aeruginosa*, and *Enterobacter* spp.) are particularly important, given their role in hospital infections and their ability to ‘escape’ the effects of antimicrobial drugs [72]. In addition to the ESKAPE pathogens, mycobacteria and NTM can contribute to persistent infections in healthcare settings [73,74].

#### 4.1.3. DUWLs and Antimicrobial Resistance

Despite numerous studies having examined the microbial contamination of DUWLs, there is still limited research on ARGs or resistant bacterial strains. One of the few studies was conducted by Vosooghi et al., who investigated NTM in DUWLs from dental centers and offices in Tehran, Iran. Both slow- and fast-growing species, including *M. chelonae*, *M. abscessus*, *M. fortuitum*, and *M. simiae*, were identified, and the drug susceptibility testing revealed high resistance to several commonly used antibiotics: *M. chelonae* and *M. abscessus* were highly resistant to ciprofloxacin, doxycycline, trimethoprim-sulfamethoxazole, and carbapenems such as imipenem and meropenem. *M. simiae* isolates showed 100% resistance to multiple antibiotics, including rifampin, isoniazid, ethambutol, and streptomycin [75]. Similarly, the antibiotic resistance pattern of *P. aeruginosa* and other *Pseudomonas* spp. in DUWLs of a public dentistry clinic in Milan, Italy, was also investigated by Tesauro et al. Out of 44 dental units, *P. aeruginosa* was found in approximately 23% of the dental units, and *Pseudomonas* spp. were identified in over 52%. Thirty percent of *P. aeruginosa* isolates and 31.8% of *Pseudomonas* spp. isolates were resistant to at least one of the six tested antibiotics (ceftazidime, piperacillin/tazobactam, meropenem, and colistin) according to antibiotic susceptibility testing. Resistance to colistin was the most common finding, which affected 22% of all tested strains. Although less common, multi-drug resistance (resistance to two or more antibiotics) was also detected [47]. Gawish et al. also studied the resistance pattern of *P. aeruginosa* from DUWLs in Alexandria, Egypt. In contrast to the Tesauro et al. study, *P. aeruginosa* was detected in only 7.5% of the samples, and all isolates were fully susceptible to the tested antibiotics, including β-lactam, carbapenem, aminoglycoside, fluoroquinolone, and polymyxin [54].

In another investigation by Vincent et al., sixteen *P. aeruginosa* isolates were obtained from DUWLs, and their genomic characteristics were evaluated using whole-genome sequencing. Some resistance genes, including β-lactam resistance (e.g., *PDC-3*, *PDC-5*, *PDC-8*) and triclosan resistance (*triC*), were absent in the *P. aeruginosa* isolates. However, some isolates showed distinct gene acquisition events, especially genes involved in membrane biogenesis, which may enhance environmental adaptation rather than antibiotic resistance. Several other isolates showed insertions in key regulatory genes, including lasR and gacS, which are essential for quorum sensing and biofilm regulation [76]. The genomic profiles of the isolates showed alterations that might affect phenotypic characteristics under selective pressure, even if they were not multi-drug-resistant. Therefore, DUWLs may be an environment where *P. aeruginosa* develops features that support persistence and, perhaps, future resistance development [76].

Alsehlawi et al. examined the antibiotic susceptibility of *L. pneumophila* isolated from DUWLs in dental clinics in Najaf, Iraq. Out of 94 water samples, they recovered nine isolates. Strikingly, all of them were completely resistant to a panel of ten antibiotics, including erythromycin, lincomycin, nitrofurantoin, gentamicin, and several β-lactams such as ampicillin, cefepime, and cefotaxime, as well as rifampin. Most isolates (88.8%) also showed resistance to amoxicillin and 77.7% were resistant to tetracycline. There was moderate resistance to doxycycline and chloramphenicol. Based on these results, nearly all isolates (88.8%) were categorized as extensively drug-resistant, with one isolate (11.1%) classified as multi-drug-resistant [77].

In a study of 50 DUWLs in Istanbul, Turkey, 74% of DUWLs showed bacterial levels higher than 200 CFU/mL, which is above American Dental Association’s standard limit [78]. Most isolates were sensitive to cefoperazone, ofloxacin, gentamicin, ciprofloxacin, and piperacillin. Nevertheless, different levels of resistance were observed, particularly among *Staphylococcus* species. All *Staphylococcus* isolates showed metronidazole resistance. *Staphylococcus sciuri* was also resistant to methicillin, rifampicin, erythromycin, and metronidazole, while *S. epidermidis* was resistant to rifampicin, chloramphenicol, erythromycin, vancomycin, and metronidazole. The risk of nosocomial infection increased due to methicillin-resistant strains [79].

In another study in Izmir, Turkey, Uzel et al. found *Pseudomonas*, *Burkholderia*, *Acinetobacter*, and *Bacillus* to be the most isolated genera out of 20 DUWLs. *Acinetobacter calcoaceticus* and *Ralstonia pickettii* showed multi-drug resistance, with limited susceptibility to piperacillin (30%), gentamicin (5%), and chloramphenicol (10%). In contrast, *Pseudomonas stutzerii* showed complete susceptibility to all tested drugs. *P. aeruginosa* showed a higher susceptibility to ceftazidime (77.3%) and meropenem (90.9%) and a lower susceptibility to ampicillin (18.2%) and tetracycline (13.6%) [80].

*S. aureus* and *S. epidermidis isolates* from 160 DUWL samples were examined by Lancellotti et al. *S. aureus* exhibited strong resistance to oxacillin (78%) and clindamycin (78%), while being most vulnerable to ciprofloxacin (97%), amoxicillin-clavulanic acid (92%), and vancomycin (91%). Similarly, *S. epidermidis* showed complete susceptibility (100%) to vancomycin and ciprofloxacin; however, resistance rates for oxacillin and clindamycin were 79% and 71%, respectively. The detection of oxacillin-resistant *staphylococci* in DUWL samples raises concern for the presence of methicillin-resistant *S. aureus* (MRSA) and methicillin-resistant *Staphylococcus epidermidis* (MRSE) in dental settings [81].

Research on AMR in DUWLs is still relatively limited. The main aim of most research on DUWL contamination is the detection of the most common microorganisms; few studies have explored the resistance patterns and the possibility of horizontal gene transfer. Since both patients and dental staff are frequently exposed to aerosols and water from DUWLs, this knowledge gap presents a growing concern, especially as AMR continues to spread globally [3].

#### 4.1.4. Water Quality and Disinfection Protocols

**Figure 4:**
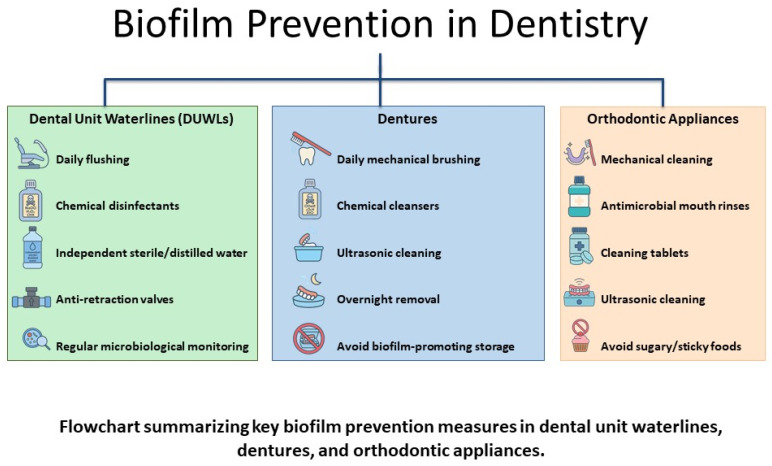
Biofilm prevention strategies in dentistry.

Regulatory authorities recommend that DUWLs should be purged for at least two minutes at the start of each clinic day and flushed for 20 to 30 s after each appointment to minimize biofilm development and lower the microbial load in treatment water [82,83]. Disinfectant tablets should be used regularly, and shock treatments should be administered as necessary in dental units with separate water reservoirs [84,85]. A long-term disinfection protocol using BRS^®^ for shock treatment, Alpron^®^ for continuous use during working hours, and Bilpron^®^ during inactivity achieved 99.8% compliance with microbiological standards in DUWLs. *Legionella* spp. and *P. aeruginosa* were consistently undetectable [86]. Both plasma sterilization and low-concentrated ozonized water have emerged as promising alternatives to traditional chemical disinfectants to reduce microbial contamination in DUWLs. However, their current use remains largely experimental, and further long-term clinical validation is needed before they can be recommended for routine implementation in dental practice (Figure 4) [87,88].

Non-surgical operations must adhere to the guidelines of Centers for Disease Control and Prevention (CDC) recommending that water contains less than 500 colony-forming units per milliliter (CFU/mL) of heterotrophic bacteria when the output water comes into direct contact with the oral cavity [89]. DUWL testing and maintenance can be done in-house by dental offices or by external laboratory services. For instance, according to the College of Dental Surgeons of Saskatchewan (CDSS), all DUWLs in Saskatchewan, Canada, should undergo testing yearly by an external laboratory; this became mandatory in 2019 [90]. However, these testing requirements and acceptable bacterial contamination levels differ regionally [51,90,91]. Furthermore, many jurisdictions, including Saskatchewan, do not require certain actions or retesting when contaminated dental unit water is found. Additionally, the frequency of DUWL testing and retesting regulations is underreported worldwide [82].

The World Health Organization’s Water Safety Plan Manual has also provided a framework for risk-based water quality management that is directly applicable to healthcare water systems, including DUWLs. Its emphasis on hazard identification, validation of control measures, and continuous operational monitoring aligns with best practices for maintaining microbiological safety in dental waterlines [92]. Moreover, the manual’s guidance on emergency response planning and verification protocols supports the development of strategies to mitigate contamination events and reduce the selective pressure that drives AMR [92]. The European Union’s shifting regulations on drinking water quality could also raise concerns. Under Council Directive 98/83/EC, the absence of *P. aeruginosa* from drinking water was mandated [93]. However, the recast Directive 2020/2184, which replaces the 1998 Directive, omits *P. aeruginosa* from its parametric requirements and instead introduces monitoring for *Legionella* spp. [94]. This regulatory shift has led to a notable gap in microbial surveillance, given the role of *P. aeruginosa* in biofilm formation within DUWL. While the inclusion of *Legionella* is a welcome addition, the exclusion of *P. aeruginosa* may create a blind spot, particularly in dental settings where *P. aeruginosa* remains a well-known biofilm-forming organism and opportunistic pathogen, especially dangerous to vulnerable patients. Its absence from EU parametric values may reduce the likelihood of its routine monitoring, potentially undermining infection prevention efforts in high-risk clinical environments [95].

### 4.2. Antimicrobial Resistance in Oral Appliances

Denture surfaces provide optimal places for bacteria and fungi to grow [96]. It is believed that acrylic-based prostheses may be a primary origin of bacterial and fungal adherence because of their porous, rough, and hydrophobic surfaces [97]. Similar findings have emerged in the study of orthodontic appliances, where biofilm formation is also influenced by ion release from metallic components [98], irregular surfaces [99], and difficult-to-clean areas [100]. Thus, AMR and the risk of oral appliance infection are major and common problems in clinical practice [101].

#### 4.2.1. Microbial Contamination and Biofilm Formation on Dentures

The most widely used and accessible material for making dentures and prosthetic teeth is polymethyl methacrylate (PMMA) [102]. Salivary pellicle production is the first step in biofilm formation on dentures. Bacteria and fungi attach to PMMA surfaces within minutes due to short- and long-range physicochemical interactions, Van der Waals forces, hydrophobic interactions, electrostatic interactions, and Brownian motion [102,103]. In their protected exopolysaccharide matrix, these initial microbial colonizers multiply and create multilayered cell clusters [104].

*Streptococcus* spp. are the most common early colonizers; their presence and metabolites change the microenvironment and create attachment sites that allow subsequent colonizers to adhere and survive. As a crucial bridge species, *Fusobacterium nucleatum* can connect early colonizers to subsequent, more harmful microbial colonizers, such as *P. gingivalis*, *Treponema* spp., *Eubacterium* spp., *Prevotella intermedia*, and *A. actinomycetemcomitans* [105]. As biofilms become more sophisticated and evolve, the oral and denture biofilm microenvironment continues to change. Obligatory anaerobic species can adhere, survive, and multiply within the biofilm when oxygen and nutrients become scarce. Advanced biofilms can contain up to 1000 different types of bacteria [106,107]. Despite the complexity of oral and denture biofilms, it has been demonstrated that *Candida* spp. may adhere to them at almost every stage of biofilm development [108]. Since *Candida. albicans* is hydrophobic, it can simply adhere to the hydrophobic PMMA [109]. According to earlier research, the level of *Candida* colonization and subsequent biofilm formation are correlated with the properties of denture acrylic surfaces [110,111]. The effects of surface roughness, permeability, and porosity on *Candida* colonization and biofilm formation on the acrylic surface of removable dentures have also been verified by some investigations [112,113].

The literature has provided clear evidence of the biofilm properties of *C. albicans* and *Staphylococcus* spp. on denture surfaces [97,114,115,116]. In one study on kidney transplant recipients wearing removable prostheses, dentures contained a substantially greater diversity and number of *Staphylococcus* strains than the pharyngeal mucosa, particularly *S. epidermidis*, *S. aureus*, and *Staphylococcus hemolyticus* [114]. Some of the pathogenic strains found in biofilm on dentures were similar to those obtained during postoperative infections [114]. In another study, Larijani et al. compared the biofilm characteristics on conventional chemical and heat-polymerized PMMA samples and CAD-CAM PMMA samples, concluding that biofilm formation and *C. albicans* adhesion on CAD-CAM samples were significantly lower compared to conventional PMMA. Moreover, scanning electron microscopy images showed that biofilms on CAD/CAM surfaces were sparser and morphologically disrupted [116]. However, De Foggi et al. found that although surface roughness increased hydrophobicity, there were no significant differences between the groups with different degrees of surface roughness in terms of biofilm formation of *C. albicans* [97].

Studies that show long-term microbial survival within denture porosities, even when the prosthesis is not in active use, further support persistent colonization [117,118]. For instance, MRSA was reported to remain viable in a dormant state within biofilms on denture surfaces, and after rehydration, its growth was reactivated [119]. Such findings strongly support that denture, primarily when poorly maintained, function as long-term microbial reservoirs capable of sustaining resistant infections.

#### 4.2.2. Common Resistant Pathogens in Dentures

As previously noted, dentures and prosthetics in the oral cavity are a significant reservoir for AMR due to biofilm formation in the porous acrylic surface [114,115,119,120,121]. Common microorganisms known for AMR include *C. albicans*, MRSA, Gram-negative bacteria such as *Klebsiella* spp. and *Pseudomonas* spp., and Gram-positive bacteria such as *Streptococcus* spp. [114,115,120,122,123,124,125,126]. Furthermore, the development of resistance to several antibiotic agents such as fluconazole, miconazole, terconazole, ketoconazole, amphotericin B, nystatin, chlorhexidine, chloramphenicol, amoxiclav, ampicillin, clindamycin, doxycycline, and methicillin has been confirmed [124,127,128,129,130]. Some risk factors have been found to contribute to AMR in denture wearers, including inadequate oral hygiene, prolonged use of dentures, systemic diseases, and prior exposure to antibiotics [5,6,11,13]. Consequently, the most effective management strategies could be antifungal/antibacterial therapies, routine disinfection of dentures, improvement of personal hygiene, and regular monitoring for microbiological infections [115,119,123,129].

*Candida* spp. isolated from denture biofilms could have different levels of resistance to antifungals. For example, a study reported that *C. albicans* had intermediate resistance to fluconazole, miconazole, and itraconazole, while non-*albicans* species had higher resistance [129]. In contrast to these results, Koga-Ito et al. found no resistant *C. albicans* on denture samples [131].

One study reported that *C. albicans* could become up to 1000 times more resistant to fluconazole and miconazole when grown in a biofilm rather than as free-floating planktonic cells, with minimum inhibitory concentration (MIC) values beyond clinical breakpoints [132]. Chandra et al. further concluded that biofilm-forming strains required 32–128× higher drug concentrations to be effective against *Candida* spp. compared to planktonic forms. Together, it could be concluded that the biofilm growth on dentures could significantly inhibit antifungal activity [130].

In several studies, *C. albicans* is often found as the primary fungal pathogen in patients with denture stomatitis, with its prevalence being 60% to 100% in denture wearers [133,134,135]. Even though most isolates have a normal response to the common antifungal drugs, resistance is increasingly reported. For instance, there was a drug resistance to fluconazole in 3.2% of *C. albicans* isolates, and the drug of choice for these patients was itraconazole in one study [124]. Aside from *C. albicans*, other *Candida* species, such as *C. glabrata* and *C. tropicalis*, were also highly resistant to fluconazole, with resistance rates approaching 18.4% [127].

In a study by Jewtuchowicz et al., the use of dental devices (both dental prostheses and orthodontic appliances) could significantly increase *Candida* carriage in subgingival biofilm. In this study, *C. albicans* was fully susceptible to fluconazole and voriconazole; however, 17% of *C. dubliniensis* isolates were resistant, and *C. guilliermondii* showed reduced fluconazole susceptibility [136].

Regarding resistant bacteria, some studies have reported MRSA and coagulase-negative *staphylococci* on denture surfaces [114,137,138,139]. In a study by Costa et al., all methicillin-resistant *Staphylococcus* were identified as *S. epidermidis*, and 94.4% carried the *mecA* gene [137]. *Staphylococcus* spp. resistance to amoxicillin, levofloxacin, cefoxitin, erythromycin, and clindamycin have also been reported by several studies [114,120,121]. Gram-negative bacteria such as *P. aeruginosa*, *K. pneumoniae*, *Enterobacter cloacae*, *E. coli*, and *Morganella morganii* were found on dentures of the elderly and showed significant resistance to ampicillin (57.4%), cephalothin (41.7%), and tetracycline (36.5%) [122].

#### 4.2.3. Risk Factors Contributing to Antimicrobial Resistance in Denture Wearers

Colonization of dentures by resistant microorganisms is highly affected by risk factors related to the prosthesis condition, patient behavior, and systemic implications. Prosthetic-related risks, such as rough and porous denture surfaces, older dentures, poor hygiene of dentures, and denture trauma due to poor fit, are highly associated with biofilm formation and microbial persistence [137,140,141,142,143,144]. Nighttime use may significantly increase microbial load on denture surfaces and the risk of denture stomatitis, which could lead to the accumulation of resistant bacteria and fungi [141,145,146,147]. Prior exposure to antibiotic medications also plays a role in the emergence of resistant strains. Even without recent use of antibiotics, past exposure may be sufficient to develop adaptive resistance in *Candida* species [148,149].

Systemic diseases, including diabetes, pneumonia, and compromised immune systems, make it easier for drug-resistant microorganisms to persist [114,129,150]. Furthermore, the oral cavity of denture wearers has lower salivary flow and provides a favorable place for fungal biofilms and various bacteria to grow [151,152,153].

#### 4.2.4. Preventing and Managing Antimicrobial Resistance in Dentures

Pharmaceutical, mechanical, and behavioral interventions are needed to address the problem of AMR among denture wearers (Figure 4).

##### Pharmacological Strategies

Topical antifungal agents such as nystatin, miconazole, amphotericin B, and clotrimazole are commonly recommended to control *Candida* overgrowth on the surfaces of dentures. These topical antifungals are typically regarded as first-line options because of their localized effect and lower risk of systemic side effects [154,155,156]. The use of systemic antifungals, such as fluconazole, can lead to resistance, and as a result, they are usually used for more severe cases. In certain situations, it may be necessary to use substitutes such as itraconazole or more recent azoles in strains resistant to fluconazole [157,158]. For biofilm-associated infections, adjuvant treatment options such as silver-based denture disinfectants, and chlorhexidine may provide additional antibacterial activity without leading to AMR [157,159,160,161].

There is growing concern over the persistence of resistant bacteria such as *S. aureus*, *P. aeruginosa*, and *K. pneumoniae* in denture biofilms. These bacteria could develop AMR to commonly used antibiotics due to frequent or inappropriate antibiotic use [120,121]. Therefore, antibacterial approaches often focus on using localized medicines such as chlorhexidine, sodium hypochlorite, or silver nanoparticles (AgNP) rather than systemic antibiotics [157,161]. The incorporation of antimicrobial agents into denture adhesives appears to be effective in improving prosthetic hygiene and eliminating biofilm formation [162]. These antimicrobial materials, such as antimicrobial drugs, nanomaterials, and phytochemical components, including plant extracts, were evaluated for their ability to inhibit microbial growth, particularly *C. albicans* [162]. Some new approaches are also being researched to target resistance mechanisms, such as the use of efflux pump inhibitors, quorum-sensing blockers, and biofilm-disrupting compounds. These emerging approaches could improve the efficacy of conventional antibiotics and prevent resistance development [163,164,165,166].

##### Mechanical and Behavioral Interventions

Producing new and well-fitting dentures, regularly brushing the denture, overnight soaking in disinfectants, avoiding overnight denture wear, and regular dental check-ups are much-needed steps to reduce the risk of biofilm formation and further colonization of resistant microorganisms [141,147,167]. Moreover, patients’ awareness of the consequences of self-medication such as the unsupervised and improper use of antibiotics or antifungals should be raised; otherwise, there would be the risk of AMR in denture-associated microorganisms [168].

Altogether, these methods reemphasize a wide-ranging, evidence-based management approach to controlling AMR in individuals with dentures. Preventing biofilm formation, proper medication selection based on susceptibility profiles, and complying with the highest level of prosthesis hygiene are all indispensable for successful infection control and resistance prevention in the long term [169].

### 4.3. Antimicrobial Resistance in Orthodontic Appliances

Orthodontic appliances, particularly fixed ones, can highly affect the oral microbiome with an increase in periodontopathic Gram-negative bacteria and cariogenic *streptococci* [170,171,172]. Moreover, wearing orthodontic devices can contribute to the development of new ecological niches, which are likely important in biofilm formation and further colonization by resistant microorganisms [173,174,175,176]. The biofilm formation on orthodontic devices is associated with other problems such as halitosis, periodontitis, gingivitis, dental caries, and white spot lesions [177,178,179].

#### 4.3.1. Biofilm Formation on Orthodontic Devices

Surface wettability, surface roughness, materials used in orthodontic devices, ion release, appliance design, and tooth position can promote microbial adhesion to orthodontic appliances and biofilm formation [99,180,181,182,183].

To bond fixed orthodontic appliances to teeth, the enamel undergoes several treatments, such as acid etching, priming, and adhesive application. Changes in surface characteristics of teeth resulting from the orthodontic bonding process may have a significant impact on the development of biofilm surrounding orthodontic devices, as increased surface wettability and surface roughness of enamel promote bacterial adhesion and biofilm formation [99].

Nickel-titanium (NiTi) arch wires are commonly used because of their outstanding mechanical characteristics, but they can lead to the corrosion of nickel ions. Nickel ions may result in the adhesion and extracellular matrix formation of *S. aureus*, which are vital for initiating biofilm formation [181]. Additionally, it has been proven that nickel can affect bacteria by functioning as a micronutrient at lower concentrations and having bacteriostatic effects at higher concentrations [184,185]. This phenomenon, which results from exposure to heavy metals, is receiving more interest in the field of antibiotic resistance and bacterial cross-adaptation. Mutations in genes encoding metal and antibiotic targets, biofilm development, efflux pump activation, and reduced membrane permeability can contribute to bacterial adaptation and resistance to heavy metals [186,187,188].

Microbial adherence and biofilm formation may be influenced by the design and material selection of orthodontic brackets and wires. According to one study, wires are more likely than brackets to develop biofilm [180]. Another study indicated that biofilm height and coverage were substantially lower in stainless steel brackets than in ceramic and gold ones [189].

In a study of patients wearing fixed orthodontic appliances, the highest biofilm accumulation was observed on the maxillary lateral incisors and maxillary canines, particularly in the gingival area and areas behind arch wires [183]. Thus, it could be argued that biofilm formation could vary among different teeth in orthodontic patients, though further research is needed to confirm this.

#### 4.3.2. Resistant Microorganisms Among Orthodontic Patients

Studies on AMR among patients receiving orthodontic treatment are limited. Patients with fixed orthodontic appliances have more diverse and resistant microbial populations in their oral cavity than those without, including higher levels of *Streptococcus* spp., *Staphylococcus* spp., *Enterobacter* spp., and *Candida* spp. In one study, approximately 74% of the isolated bacterial strains from orthodontic patients were resistant to at least one antimicrobial (ampicillin, ampicillin/sulbactam, benzylpenicillin, cefoxitin, cefuroxime, cefuroxime axetil, and clindamycin) [175]. These findings concurred with those of Abbas et al., who showed that *S. aureus* was more frequently isolated from patients with orthodontic appliances (57%) compared to non-wearers (43%). They also concluded that NiTi and stainless steel wires were associated with increased antibiotic resistance, particularly to bacitracin, ceftazidime, amoxicillin-clavulanic acid, and erythromycin [101]. Similarly, higher AMR and the increased ability for adhesion and biofilm formation on NiTi arch wires among *S. aureus* strains adapted to moderate nickel concentrations (250 µg/mL) were reported by Pelvic et al. [181]. In another investigation, a significant correlation between recent fixed orthodontic treatment and the presence of the *agr* gene in isolates of *Streptococcus mutans* was found. However, no link was observed between the analyzed genes (*mecA*, *pvl*, or *coa*) and using removable braces [190].

In one study, resistant *Enterococcus* and *E. coli* were observed in fixed orthodontic appliance wearers with poor oral hygiene, in contrast to the healthy controls without orthodontic appliances. They found resistance to erythromycin, tetracycline, and kanamycin, with some bacteria carrying multiple resistance genes, including *erm*(B), *tet*(L), and *aph*(3′)-IIIa [191]. Jewtuchowicz et al. reported a higher level of *Candida* spp. in subgingival biofilm of patients using orthodontic appliances compared to non-users. They also observed that *C. dubliniensis* and *C. guilliermondii* had some resistance to azoles, including fluconazole and voriconazole [136].

Taken together, these studies suggest that treatment with fixed orthodontic appliances can increase the resilient bacterial populations within oral biofilms (Table 1).

#### 4.3.3. Antimicrobial Resistance Prevention in Orthodontic Device Users

Regular oral hygiene practices, avoiding unnecessary antibiotic prescriptions, scheduling routine professional cleanings to disrupt biofilm maturation, and the integration of antimicrobial technologies are all important components of a multifaceted approach to prevent AMR in orthodontic patients [175,183,191,192,193,194]. Patients should be instructed on maintaining good oral hygiene, which includes regular brushing, flossing, and using antimicrobial mouthwashes (Figure 4).

Dental professionals should prescribe antibiotics only when clinically necessary and take steps toward AMS. Additionally, antimicrobial coatings, such as AgNP coatings on brackets and wires, have demonstrated potential in decreasing microbial colonization and biofilm development [195,196]. Other promising materials for orthodontic wire treatment include Teflon (PTFE), zirconium dioxide (ZrO_2_), and composite resins, which have been shown to reduce ion release and bacterial adhesion [197,198,199].

**Figure 5:**
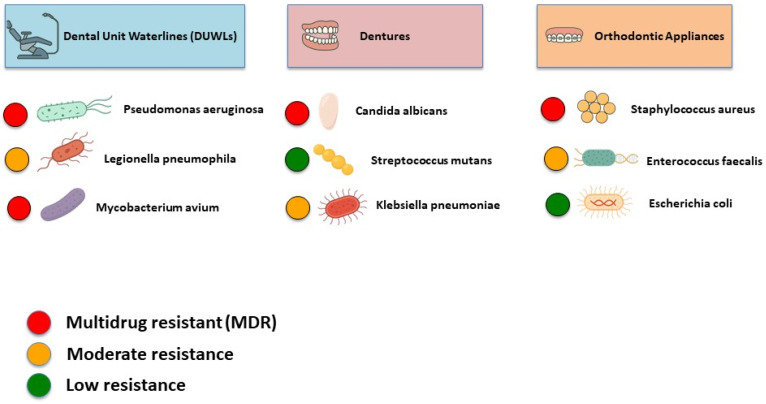
AMR in key dental reservoirs: DUWLs, dentures, and orthodontic appliances.

Table 2 and Figure 5 provide an overview of the common pathogens isolated from all three discussed reservoirs, along with their reported AMR profiles.

## 5. Recommendations for AMS Implementation in Dentistry

Effective employment of AMS in dentistry requires a broad and context-specific approach that addresses both clinical practice and behavioral change. Recent evidence emphasizes raising awareness among dental teams and patients about the risk of unnecessary antibiotic use [20]. Antimicrobials should be prescribed in dentistry only when clinically indicated [200]. Therapeutic antibiotics must be reserved for bacterial infections with systemic involvement (such as fever, malaise, lymphadenitis, or rapidly spreading cellulitis) and avoided for localized infections manageable with operative measures alone [13,201,202]. Prophylactic use should be restricted to high-risk patients, such as those with specific cardiac conditions predisposing to infective endocarditis or certain immunocompromised states [203,204]. Prescribers should choose the narrowest effective spectrum, the shortest effective course, and appropriate dosing, and provide clear instructions to minimize unnecessary exposure and reduce AMR [205].

Integrating AMS principles into undergraduate and continuing education and promoting practices with a culture of stewardship should also be considered [20,206,207]. Distributing guidelines alone rarely leads to meaningful changes in prescribing habits. Long-lasting progress is more likely when these guidelines are combined with regular audits, constructive feedback, and targeted educational initiatives for dental professionals [208]. Collaboration with pharmacists, infectious disease specialists, and other healthcare professionals can strengthen AMS strategies, and involving educators from outside dentistry during undergraduate training has been shown to broaden perspectives and promote interprofessional teamwork [207]. To mitigate the need for antibiotics as a substitute for treatment, it is crucial to ensure that patients have access to timely definitive dental care and to adjust AMS interventions to local prescribing patterns, resources, and cultural factors [209]. Embedding AMS resources, such as prescribing recommendations, into dental software, and using patient education tools (posters, videos, and leaflets) can further reinforce best practice [206].

Novel clinical approaches can minimize the use of unnecessary antibiotics in dentistry. For example, the use of platelet-rich fibrin (PRF) in oral surgery has been shown to enhance soft tissue healing, reduce postoperative complications, and lower infection rates, which could decrease the reliance on prophylactic or therapeutic antibiotics. Integrating such adjunctive techniques into routine practice may support AMS goals by addressing infection prevention at the procedural level [210].

Finally, effective AMS in dentistry should be embedded within broader public health strategies that aim to reduce the spread of AMR. Preventive measures, such as maintaining good oral hygiene, ensuring timely access to dental care, and enforcing rigorous infection prevention protocols, reduce the need for antibiotics and protect their long-term efficacy. By integrating public health initiatives with evidence-based prescribing, dental professionals can help prevent the emergence and transmission of resistant pathogens and contribute to global efforts to improve antibiotic effectiveness.

## 6. Conclusions

In conclusion, a wide range of resistant microorganisms have been detected in DUWLs, as well as on the surfaces of dentures and orthodontic appliances. Many of these pathogens are multi-drug resistant and can persist in biofilms for extended periods, increasing the risk of transmission in dental settings. Although resistance genes have been identified within these biofilm-associated communities, our understanding of how these genes are exchanged, through mechanisms such as transformation, transduction, and conjugation, remains limited. Future research should focus on elucidating these genetic transfer processes and identifying the environmental and clinical conditions that facilitate gene exchange.

Longitudinal and in situ studies are also essential to monitor resistance development over time under real-world conditions. Given the routine exposure of both patients and dental professionals to aerosolized particles, particularly during procedures involving DUWLs and contaminated appliances, the presence of resistant pathogens poses a serious clinical and public health concern. To address this, more stringent infection control protocols and AMS programs specifically designed for dental environments must be developed and consistently implemented. Standardizing AMR surveillance in dentistry, improving patient education, and integrating AMR-focused content into dental curricula and continuing professional development are also critical to reducing the spread of resistance and safeguarding both oral and systemic health.

## Figures and Tables

**Table 1:** Microbial populations associated with braces and retainers.

Feature	Braces	Retainers	References
Dominant Microorganisms	Higher abundance of *Streptococcus mutans*, *Lactobacillus* spp., and anaerobes	*Candida* spp. more prevalent, alongside *S. mutans* and mixed anaerobes	[101,175,181,190,191]
Bacterial Load	Significantly increased compared to non-orthodontic patients	Varies by retainer type; removable retainers may have lower loads than fixed ones	[101,175,181,190,191]
Biofilm Characteristics	Thicker and more mature biofilms due to brackets and wires creating retention sites	Biofilms can form on both acrylic and wire components; removable types allow better cleaning	[101,175,181,190,191]
Health Implications	Increased risk of enamel demineralization, white spot lesions, and gingivitis	Potential for oral candidiasis, increased caries risk, and periodontal inflammation	[101,175,181,190,191]

**Table 2:** The most common pathogens found in DUWLs, dentures, and orthodontic appliances, and their resistance patterns.

Pathogen	Location	AMR Profile	Reference
*M. chelonae*, *M. abscessus*, *M. lentiflavum*, *M. fortuitum*, *M. kansasii*, *M. simiae*	DUWLs	High resistance rates were observed to trimethoprim-sulfamethoxazole, doxycycline, imipenem, meropenem, and ciprofloxacin. Moderate resistance to moxifloxacin, cefoxitin, and clarithromycin was observed. In contrast, lower resistance rates were noted for streptomycin, ethambutol, rifampin, and isoniazid.	[75]
*P. aeruginosa*	DUWLs	30% of strains were resistant. The most common resistance was to colistin (21.4%), followed by piperacillin (5.7%) and ceftazidime (2.9%). Multi-drug resistance included combinations such as levofloxacin-netilmicin-colistin and piperacillin-ceftazidime-colistin.	[47]
*P. aeruginosa*	DUWLs	All isolates were sensitive to all tested antibiotics, including piperacillin, tazobactam, ceftazidime, cefepime, aztreonam, imipenem, meropenem, colistin, polymyxin B, gentamicin, tobramycin, amikacin, and ciprofloxacin.	[54]
*P. aeruginosa*	DUWLs	Isolates lacked several ARGs commonly found in clinical strains, such as *PDC-3*, *PDC-5*, *PDC-8*, and *triC*. The most basal isolates carried additional resistance-related genes, including *golS*, *mdsA*, *ceoB*, and *mdsC*.	[76]
*L. pneumophila*	DUWLs	The isolates were 100% resistant to multiple antibiotics, including erythromycin, lincomycin, gentamicin, and several β-lactams. High resistance was also seen to amoxicillin (88.8%), tetracycline (77.7%), and doxycycline and chloramphenicol (55.5%). Amikacin showed the lowest resistance.	[77]
*P. aeruginosa* *P. fluorescens* *P. lutea* *P. putida* *Staphylococcus* spp.	DUWLs	*Pseudomonas* spp. were susceptible to cefoperazone, ofloxacin, gentamicin, ciprofloxacin, and piperacillin but resistant to metronidazole and often to rifampicin and erythromycin. *Staphylococcus* spp. were also susceptible to those antibiotics, but all were resistant to metronidazole. Some strains, such as *S. sciuri* and *S. epidermidis*, were methicillin-resistant and multi-drug-resistant.	[79]
*Pseudomonas* spp. *Burkholderia* spp.	DUWLs	*Pseudomonas* spp. were highly susceptible to ceftazidime, meropenem, and ofloxacin but showed strong resistance to ampicillin. *P. aeruginosa* had only 18.2% susceptibility to ampicillin. *Burkholderia* spp. showed good susceptibility to ceftazidime and meropenem. However, they were highly resistant to ampicillin, gentamicin, and chloramphenicol.	[80]
*S. aureus* *S. epidermidis*	DUWLs	*S. aureus* and *S. epidermidis* showed high resistance to oxacillin and clindamycin. Both had moderate resistance to ampicillin, amoxicillin, and azithromycin. Ciprofloxacin and vancomycin were effective against all isolates. *S. aureus* was slightly more susceptible overall than *S. epidermidis*.	[81]
*Streptococcus* spp. Coagulase-negative Staphylococci, *S. aureus* *E. coli* *K. pneumoniae* *P. aeruginosa*	Dentures	Amikacin, nalidixic acid, and ciprofloxacin showed the highest sensitivity pattern, while cefixime and amoxicillin/clavulanic acid were the most resistant antibiotics. *E. coli* exhibited complete sensitivity to all tested antibiotics, while *P. aeruginosa* showed multi-drug resistance, especially against cotrimoxazole and nitrofurantoin.	[139]
*Viridans streptococci*, *S. aureus* *K. pneumoniae*, *E. coli*	Dentures	Viridans streptococci showed the highest resistance to amoxicillin-clavulanic acid (40%) and methicillin (35%), while *S. aureus* had 25% resistance to both amoxicillin and methicillin. *K. pneumoniae* exhibited the highest resistance to amoxicillin-clavulanic acid (40%) and 30% resistance to cefotaxime, gentamicin, and doxycycline; E. coli showed 50% resistance to amoxicillin-clavulanic acid and 30% to multiple other antibiotics. The presence of dense biofilms and extracellular matrices observed via SEM suggested these structural features contributed to the observed multi-drug resistance.	[120]
*Staphylococcus* spp.	Dentures	Isolates showed high resistance to penicillin (91.6%), fosfomycin (87.5%), and cefoxitin (62.5%), indicating widespread methicillin resistance. Moderate resistance was observed to erythromycin (55.5%), tetracycline (43%), and clindamycin (38.8%). Resistance to gentamicin (16.6%) and trimethoprim/sulfamethoxazole (25%) was lower, while all isolates remained susceptible to linezolid and vancomycin. Nearly half (48.6%) of the isolates were multi-drug resistant.	[114]
*S. aureus*	Dentures	MRSA was found on 1% of outpatients’ and 12% of inpatients’ dentures, with most isolates identified as the highly resistant EMRSA-15 strain. All MRSA isolates were resistant to β-lactam antibiotics, including methicillin, oxacillin, and cefoxitin.	[138]
*Staphylococcus* spp.	Dentures	*S. aureus* and coagulase-negative Staphylococci were isolated. Colonization by MRSE was significantly higher (42.9%) compared to non-denture wearers (16.9%). All MRSE isolates were identified as *S. epidermidis*, with 94.4% carrying the *mecA* gene. MRSE strains exhibited greater resistance to antibiotics such as mupirocin while remaining susceptible to dalfopristin/quinupristin and linezolid.	[137]
*P. aeruginosa*, *Klebsiella* spp. *Enterobacter* spp. *Enterococcus* spp. *Staphylococcus* spp. *Streptococcus* spp.	Dentures	Resistance was high to ampicillin, amoxicillin, cephalothin, and tetracycline. Carbapenems (imipenem and meropenem) and rifampin were the most effective antibiotics, although some resistance still occurred.	[121]
*E. faecalis* *E. faecium* *E. coli* *M. morganii* *P. aeruginosa* *Klebsiella* spp.	Dentures	High resistance was observed to ampicillin (57.4%) and tetracycline (36.5%), with notable β-lactamase production in 41.2% of isolates, particularly *blaTEM*, *blaSHV*, and *blaCTX-M* genes in Gram-negative rods. Tetracycline resistance genes were diverse, with *tet*(*A*) and *tet*(*B*) common in Gram-negatives and *tet*(*K*) and *tet*(*M*) dominant in enterococci.	[122]
*C. albicans* and non-albicans *Candida* isolates	Dentures	All *C. albicans* isolates were susceptible to amphotericin B, with only 5.6% and 7.0% showing resistance to fluconazole and itraconazole, respectively. Non-*albicans* species showed higher resistance: 18.4% were resistant to fluconazole and 10.2% to itraconazole, though around 80% remained susceptible to these drugs. Amphotericin B and 5-fluorocytosine were the most effective agents against all *Candida* isolates.	[127]
*C. albicans*	Dentures	No antifungal resistance was observed in any of the isolates.	[131]
*C. albicans* non-albicans *Candida* isolates	Dentures	Denture use was associated with a higher oral yeast load and increased resistance to antifungal agents, particularly miconazole and 5-fluorocytosine. Non-*albicans* species showed higher resistance rates to fluconazole (29%), miconazole (35%), and ketoconazole (23%) compared to *C. albicans*.	[129]
*C. albicans*	Dentures	*C. albicans* were highly resistant to fluconazole and miconazole, showing MICs ≥ 256 mg/L, which was over 1000 times higher than for planktonic cells (MIC 0.25 mg/L). Chlorhexidine was more effective, with biofilm inhibition at concentrations 8 times higher than its planktonic MIC (0.3% vs. 0.04%). Young biofilms (2–6 h) were significantly more susceptible to all agents, especially miconazole and chlorhexidine, compared to mature (72 h) biofilms.	[132]
*Staphylococcus* spp.	Dentures	*S. epidermidis*, *S. hominis*, and *S. xylosus* were found on dentures, and methicillin-sensitive *S. aureus* was recovered from only one denture, and MRSA was found on two partial dentures. No MRSA was recovered from complete dentures.	[126]
*C. albicans*	Dentures	Biofilm-grown *C. albicans* were significantly more resistant to amphotericin B, nystatin, chlorhexidine, and fluconazole compared to planktonic cells. Biofilm-associated *C. albicans* required up to 128–256 times higher concentrations to achieve the same metabolic inhibition.	[130]
*C. albicans* non-albicans *Candida* isolates	Dentures	Fluconazole resistance was observed in 3.2% of isolates, mainly involving *C. tropicalis* and one strain of *C. albicans*. Only two of the fluconazole-resistant strains also showed resistance to itraconazole. Clinical cure rates were high with fluconazole (97%), and itraconazole was effective in all fluconazole-resistant cases.	[124]
Methicillin-resistant *S. aureus*	Dentures	All isolates were confirmed to carry the *mecA* gene and had high oxacillin MICs (96–256 µg/mL).	[119]
*C. albicans* *Streptococcus* spp. *Staphylococcus* spp. *Lactobacillus* spp.	Dentures	Antifungal resistance was noted in *Candida* strains, with 3 resistant to amphotericin B, 4 to nystatin, and 1 resistant to both. Nystatin was the most effective treatment, though some cases required combination therapies due to drug resistance.	[128]
*S. aureus*	Orthodontic Appliances	100% of isolates were resistant to oxacillin and cefoxitin, classifying them as MRSA. 61% were resistant to vancomycin. Genetic characterization revealed nuc gene (100%), *mecA* gene (55.6%), *pvl* gene (50%), *agr* gene (33.3%), and *coa* gene (55.6%). *mecA*/*coa* genes showed significant associations with male gender and smoking, as well as with antibiotic use and dietary patterns. The *pvl* gene showed association with mouth rinse use and buccal cavity inflammation.	[190]
*S. aureus* *S. hominis Enterobacter cloacae* complex, *Klebsiella oxytoca* *P. aeruginosa*	Orthodontic Appliances	74% of isolated bacteria showed resistance. The most common resistance was to beta-lactam antibiotics (ampicillin, cefoxitin, and cefuroxime), followed by erythromycin, clindamycin, and tetracycline.	[175]
*S. aureus*	Orthodontic Appliances	Bacteria adapted to 250 µg/mL Ni^2+^ showed the highest adhesion, biofilm biomass, and resistance to some antibiotics. Bacteria adapted to 62.5–250 µg/mL Ni^2+^ showed increased resistance to gentamicin, benzylpenicillin, rifampicin, trimethoprim-sulfamethoxazole, moxifloxacin, cefoxitin, and linezolid. Strains adapted to higher Ni^2+^ concentrations (500–1000 µg/mL) were more susceptible. Increased resistance was associated with lower nickel adaptation, while higher adaptation reduced resistance and biofilm adhesion.	[181]
*S. aureus*	Orthodontic Appliances	Increased resistance was observed in isolates treated with NiTi and SS wires. Resistance in both original and mutated isolates to ceftazidime. Some mutated isolates showed resistance to bacitracin, erythromycin, ogmentin, and amikacin, especially after prolonged incubation (48–96 h). Most isolates remained sensitive to cefotaxime. Mutation induced by orthodontic wire exposure increased resistance over time.	[101]
*E. faecalis* *E. faecium* and *E. coli*	Orthodontic Appliances	Among the *Enterococcus* isolates, resistance was detected as follows: 100% to erythromycin (associated with the *erm*(*B*) gene), 75% to kanamycin (*aph*(3′)-*IIIa0*, 50% to tetracycline (*tet*(*L*) with or without *tet*(*M*)), 37% to streptomycin (*ant*(6)-*Ia*), and 12% to chloramphenicol (*catA*). One *E. coli* isolate exhibited a multi-drug resistant phenotype, carrying five resistance genes along with both class 1 and class 2 integrons. All *Enterococcus* strains produced gelatinase, and four harbored genes for enterocins L50A/B and P. The *esp* virulence gene was identified in one multi-drug resistant *E. faecalis* isolate.	[191]
*C. albicans* and non-albicans *Candida* isolates	Orthodontic Appliances and Dentures	All *C. albicans*, *C. parapsilosis*, *C. tropicalis*, and *C. sake* isolates were fully susceptible to both fluconazole and voriconazole. Among the non-*albicans* species, one *C. dubliniensis* isolate (17%) was resistant to both antifungals, while all *C. guilliermondii* were fully susceptible to voriconazole.	[136]

DUWLs: dental unit waterlines, ARGs: antibiotic resistance genes, SEM: scanning electron microscopy, MRSA: methicillin-resistant *Staphylococcus aureus*, MRSE: methicillin-resistant Staphylococcus epidermidis, NiTi: Nickel-titanium, SS: stainless steel.

## Data Availability

Not applicable.
